# Quality of Life in Caregivers of Cancer Patients: A Literature Review

**DOI:** 10.3390/ijerph20021570

**Published:** 2023-01-15

**Authors:** María Dolores Guerra-Martín, María Del Rocío Casado-Espinosa, Yelena Gavira-López, Cristina Holgado-Castro, Inmaculada López-Latorre, Álvaro Borrallo-Riego

**Affiliations:** 1 Department of Nursing, Faculty of Nursing, Physiotherapy and Podiatry, University of Seville, 41009 Seville, Spain; 2 Faculty of Nursing, Physiotherapy and Podiatry, University of Seville, 41009 Seville, Spain; 3 Valme Hospital, Andalusian Health Service, 41014 Seville, Spain; 4 Quirónsalud Maternal and Child Hospital, 41013 Seville, Spain

**Keywords:** caregivers, neoplasms, nursing care, quality of life, social problems

## Abstract

(1) Background: Cancer constitutes one of the principal causes of morbi-mortality in the world and generates an important loss of patients’ self-sufficiency. People who are their caregivers usually become the main care providers, which impacts their quality of life; (2) Aim: Analyze the different problems (physical, emotional, social, and financial) faced by people who are caregivers of adults with cancer and describe the strategies required to improve their quality of life; (3) Method: A literature review was conducted on the following database: PubMed, Cinahl, PsycINFO, and Scopus. The following eligibility criteria were specified: (a) research studies of quantitative, qualitative, or mixed methods, (b) consistent with objective, and (c) published in the English language or Spanish during the last five years; (4) Results: 36 studies were selected from those found in the literature. Regarding the problems mentioned: eight studies described physical issues, 26 emotional effects, 10 social implications, and seven financial strains. Twenty-eight studies described strategies to improve the quality of life of caregivers; (5) Conclusions: Caregivers are usually women around the age of 50. Problems faced are mostly emotional in nature, followed by social, physical, and financial ones. In order to cope with this burden, there are some strategies that can be developed to help to build skills to manage both the disease and the impact derived from it, therefore improving their quality of life.

## 1. Introduction

Cancer is considered the disease of the 21st century, as it is a leading cause of mortality and morbidity in the world [1]. According to the WHO, cancer is one of the leading causes of death through disease and its cure is still unsolved despite the advances in diagnosis and treatment [2]. The International Agency for Research on Cancer estimated that in the year 2020, 18.1 million cases of cancer were diagnosed worldwide, and this number will increase in the following two decades, reaching 27 million [3].

Cancer, as other chronic diseases, has numerous sequelae and an important limitation of self-sufficiency, thus requiring support from other people, usually from their immediate circle. Those people are known as informal or non-professional carers. The role of these caregivers is key in aiding and supporting cancer patients, however, they may experience a significant burden at numerous levels, causing an impact in their quality of life [4,5].

According to different authors, there is an inverse correlation between burden and quality of life in the caregivers of patients with cancer [6]. At the same time, being the caregiver of a care-dependent person implies 86% risk of suffering anxiety, and 65% of suffering depression [7]. 

Diverse studies have profiled non-professional caregivers, concluding that they are mostly women, aged 45 to 65, homemakers, and the daughter or spouse of the patient [8,9]. Besides, 39% of caregivers have no education and the average time devoted to provide care is 10.9 h per day [5]. 

Cancer causes devastation at physical, emotional, and social levels to people who suffer it and their families [10]. Epidemiologic data show that 40–50% of people diagnosed with cancer suffer from a high degree of physical and psychosocial pain along the whole oncological process, where 30% of patients asked for professional support. Family caregivers undergo an adaptation and acceptance process with regard to the disease, facing numerous tasks with high level of stress, which result in physical, emotional, social, and financial draining, therefore affecting their quality of life [11,12].

Given the significant exhaustion of informal caregivers, studies regarding their quality of life have gained importance in the last few years within the health sphere [13]. The concept quality of life is greatly influenced by the social context experienced by the individual and the relationship this person establishes with everything that surrounds her or him [14]. In this sense, the WHO has defined the term quality of life as the perception that each individual has of her/his own position in the cultural context and system of values she/he is experiencing, and this is closely related to this person’s goals, expectations, life standards, and concerns [15].

Different authors have described both dimensions and indicators embodied by the quality of life, as follows: 1. Physical well-being dimension, being its indicators health, nutrition, leisure, mobility, and the capacity to develop daily life basic activities. 2. Emotional dimension, comprising indicators, such as happiness, sense of security, spirituality, self-concept, and absence of stress or fear. 3. Social dimension, with indicators based on interpersonal relationships, such as family, friends, received support, social status, work environment, role in the community, etcetera. 4. Dimension of material well-being, including indicators related to the individual’s rights, socioeconomic status, safety, and employment [16,17,18,19].

In this respect, the objective of this review is focused on analyzing both problems and improvement strategies which are associated with the quality of life of people who are caregivers of adults with cancer.

## 2. Materials and Methods

### 2.1. Study Design and Search Strategy

A literature review was conducted on PudMed, Cinahl, PsycINFO, and Scopus during the months of February and May 2022. The studies’ search strategy was defined by consensus between the authors in order to avoid bias for not including relevant studies [20], namely: [(“Quality of Life” OR “Social Problem”) AND Neoplasm AND (Caregivers OR “Nursing Care”)].

### 2.2. Eligibility Criteria

Eligibility criteria for studies were presented with the greatest transparency and clarity in order to control selection bias [21]. Those eligibility criteria were: (1) Research studies following a quantitative, qualitative, or mixed method. (2) Regarding the issues associated to people who provide care to adults with cancer. (3) People aged 18 or older. (4) Published in the English or Spanish language during the past five years (2017–2022).

### 2.3. Selection of Articles

Studies were selected in four stages. The first of them consisted in the search and localization of studies on the database through the selected search strategy. The second stage comprised the elimination of duplicates. The third stage was devoted to titles, keywords, and abstracts reading of the selected studies, choosing those consistent with the review subject and according to the eligibility criteria. A fourth stage consisted of a critical reading of the full texts of the selected articles.

### 2.4. Data Analysis

Evidence drawn from selected studies included: 1. Author/s and year. 2. Objective. 3. Country/Period of the study. 4. Type of study/Instrument/Sample. 5. Main findings. Data were collected in a table following the recommendations of author Del Pino et al. [20].

## 3. Results

### 3.1. Description of the Studies

**Figure 1:**
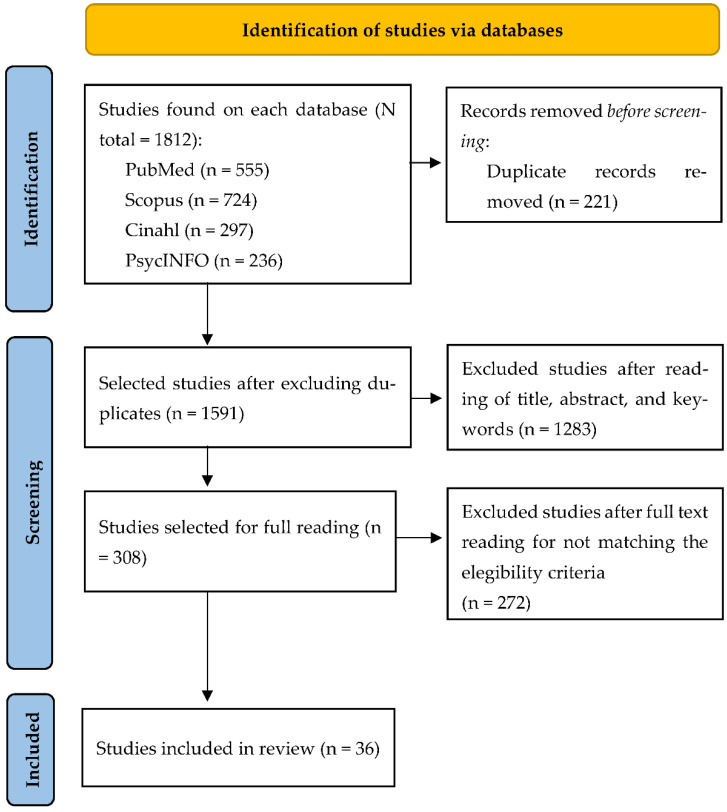
Selection of studies flowchart.

Of the 1812 studies initially identified, 36 were selected as being consistent with the review objective and eligibility criteria (Figure 1). 

With regard to their typology, 28 studies followed a quantitative research method. Twenty-two of them were descriptive, exploratory, or based on observation; four were randomized controlled trials; one was an experimental controlled trial; and one was a case–control study. In the remaining studies, seven followed a qualitative research method and one, a mixed method. Specific characteristics of these studies are detailed in Table 1.

### 3.2. Study Outcome

#### 3.2.1. Characteristics of the Sample of Caregivers of Adults with Cancer

The total sample comprised 7663 people caregivers with an average age of 54.42 years. However, it must be highlighted that 10 studies did not describe average age [22,23,24,25,26,27,28,29,30,31]. With regard to sex, 66.72% of caregivers were women. As for family bonds, 15 studies described these caregivers as being mostly spouses or daughters/sons of the patients with cancer, usually sharing the same home [22,23,25,26,27,28,30,32,33,34,35,36,37,38,39].

#### 3.2.2. Problems (Physical, Emotional, Social, and Financial) of People Who Are Caregivers of Adults with Cancer

In relation to physical problems, eight studies [25,34,37,40,41,42,43,44] described that care work implies a greater risk for caregivers of suffering physical morbidity with considerable wear, which in fact increases as the care burden continues over time. It is also observable that physical and health problems are more frequent in female caregivers, and physical damage results in a worse quality of life perceived by caregivers. 

At an emotional level, 26 studies [22,23,25,26,27,28,29,30,33,34,35,36,38,40,41,42,43,44,45,46,47,48,49,50,51,52] alluded to the emotional and psychological problems suffered by caregivers, highlighting anxiety, depression, feelings of grief and distress, and high levels of stress. Therefore, showing that, the longer the care was needed over time, the greater the tension and emotional burden, resulting in worse quality of life.

With regard to social aspects, 10 studies [24,25,27,31,32,41,49,53,54,55] described how the patients’ disease process has affected the social interaction of caregivers, who manifest a great difficulty in reconciling their role as caregivers and the development of other daily activities, highlighting the deterioration of working conditions. The studies conclude that less social support is related to worse quality of life.

In the economic sphere, 10 studies [25,27,39,44,53,56,57] described the financial problems of people caregivers, resulting from the costs associated with the disease, the lack of financial support of other family members, or the difficulty of reconciling their role as caregivers with their work activity. The studies show that caregivers with higher income levels perceive a better quality of life.

#### 3.2.3. Strategies to Improve the Quality of Life of Caregivers of Adults with Cancer

Among the strategies described, 10 studies [28,29,31,32,36,39,41,50,54,57] emphasized the need of enhancing social support by means of reinforcing the support networks and facilitating access to support groups and associations. Thirteen studies [23,24,27,30,32,35,37,38,45,46,47,49,54] indicated the need of developing coping strategies to improve the management and control of both the situation and the emotional strain, and also to help increase training in caregivers, thus improving their self-efficacy while reducing the emotional impact. One study [53] included the completion of breathing exercises and counseling sessions to reduce emotional strain. One study [34] suggested exercising practice to improve the perceived health levels on the part of caregivers. One study [39] pointed out the need of providing financial support to caregivers in order to improve their well-being. Four studies [22,24,44,48] highlighted the need of training properly health professionals on existential and spiritual distress management, as well as the need to identify groups at risk and reassigning tasks among all family members. One study [26] described that health professionals require cultural awareness in order to properly meet the needs of caregivers. 

**Table 1:** Characteristics of the selected studies.

Author/s and Year	Objective	Country/Period	Type of Study/Instrument/Sample	Main Findings
Arias-Rojas et al., 2022 [32]	To adapt and validate a LatAm-Spanish version of the QOLLTI-F scale applied to family caregivers of patients in palliative care.	Colombia/May to November 2019	Quantitative study (descriptive) Instrument: QOLLTI-F. Sample: 208 caregivers (44 men and 164 women, average age: 49.67 years)	Deterioration of social interaction, medical care, and meaning of life. Caregivers are mostly women, daughters, or spouses/partners. They showed low quality of life, being the emotional dimension the most affected, with a significant increase in anxiety and depression levels. Strategy: Consider essential to detect exhaustion in the role of caregiver. Social support is required, besides developing strategies which allow a better management and control of the situation. This coping mechanism must be also addressed to reduce emotional impact.
Benites et al., 2022 [22]	Understand the spiritual and existential experiences of family caregivers of patients with advanced cancer facing end of life in Brazil.	Brazil/June 2018–March 2019	Qualitative study through interviews Sample: 16 caregivers (3 men and 13 women, average age: non-described)	Family caregivers experienced existential and spiritual distress in the form of guilt, emotional repression, and loneliness when facing end of life. Strategy: It is required to provide training to health professional on the management of existential and spiritual distress of caregivers.
Sadigh et al., 2022 [56]	(1) Validate the instrument Comprehensive Score for Financial Toxicity (COST) modified in non-professional caregivers of patients with cancer, (2) Identify factors associated with financial toxicity both in patients and caregivers, and (3) Evaluate the impact of financial toxicity in specific aspects of both patients and caregivers.	USA/January–September 2019	Quantitative study (observational cross-sectional) Instrument: questionnaire COST for non-professional caregivers, FACT-G, CarGOQoL, Brief-POMS, and MCSDS. Sample: 100 caregivers (45 men and 55 women, average age: 56.6 years)	Caregiving generates financial toxicity for caregivers due to costs growth and income loss. This financial toxicity may be even increase when care provision extends over time and the burden on the caregiver is greater. Increase of financial toxicity of caregiver correlates with higher care non-adherence in patients, increased lifestyle-altering behaviors, and low quality of life.
Kassir et al., 2021 [36]	Evaluate if psychosocial distress among caregivers of patients with head and neck cancer is associated with a difference in how caregivers and their patients perceive patients’ quality of life.	USA/August 2019–April 2020	Quantitative study (cross-sectional) Instrument: UWQOL, PHQ-8, and GAD-7 Sample: 47 caregivers (14 men and 33 women, average age: 62.6 years)	Caregivers were predominantly women and spouses or partners of the patient, with a dedication to care exceeding nine hours/week. Most caregivers had spent more than one year providing care. Caregivers perceived their quality of life in a more negative way than patients with cancer, showing considerably greater levels of distress. Strategy: Facilitate meetings between patients and caregivers which allowed them to express their thoughts and feelings about the disease process and thus share how their own well-being was affected.
Lim et al., 2021 [40]	Examine the lifestyle of caregivers of family members with cancer, particularly the meaning of leisure and focusing on their difficulties and the role of leisure.	South Korea/Period: Non-described	Qualitative study through interviews. Sample: −10 caregivers (1 man and 9 women, average age: 44.7 years)	Caregivers showed high levels of stress and both psychological and physical conflicts, resulting in poor quality of life. Caregivers described that leisure was necessary and could improve their quality of life; however, indicated feeling of guilt when engaging in personal activities.
Nayak and George, 2021 [53]	Determine the effectiveness of multicomponent intervention on quality of life of family caregivers of cancer patients.	India/November 2016–February 2019	Quantitative study (case and control) Instrument: QOLLTI-F. Sample: 200 family caregivers (78 men and 122 women, average age: 41 years)	Non-professional caregivers had to face financial problems due to the disease condition of their loved one. Lack of financial support received from other family members influenced their low quality of life. Likewise, caregivers faced difficulties in their work life and family relations. Strategy: Yoga breathing exercises, counselling and training conducted by yoga therapists. The program resulted effective in improving the quality of life of caregivers.
Abbasi et al., 2020 [57]	Determine the relation between care burden and quality of life of caregivers of patients with cancer in a referral hospital in Iran.	Iran/2018	Quantitative study (cross-sectional) Instrument: SF-36, Caregiver Burden Inventory. Sample: 154 family caregivers (46 men and 108 women, average age: 41.30 years)	Increase of care burden resulted in a significant decrease of quality of life of caregivers. Besides, it was found that married caregivers had better quality of life than single ones, and caregivers with mid-high incomes reported a higher quality of life in comparison with those having a lower income. Strategy: It is recommended to reinforce and expand the support networks for caregivers of patients with cancer. Facilitate access to self-help associations.
El-Jawahri et al., 2020 [45]	Evaluate viability and preliminary efficacy of multimodal psychosocial intervention for family caregivers of patients undergoing hematopoietic stem cell transplantation designed to improve quality of life, burden, mood, and self-efficacy of caregivers.	USA/December 2017–April 2019	Randomized controlled trial Instrument: CarGOQOL, CRA, HADS, CASE-T, and MOCS. Sample: 100 caregivers (28 men and 72 women, average age: 61 years)	Caregivers of patients experience psychological problems (depression and anxiety) and has an overload of care throughout the care process. Strategy: Psychosocial multimodal conducted by a team of oncologists and psychologists. Caregivers who were randomized to the intervention group reported better quality of life, less overload, less anxiety and depression symptoms, more self-efficacy and coping skills when compared to the control group.
Halkett et al., 2020 [35]	Explore the lived experienced by caregivers of patients diagnosed with head and neck cancer.	Australia/November 2018–June 2019	Qualitative study through interviews. Sample: 20 caregivers (1 man and 19 women, average age: 56 years)	Caregivers showed high levels of distress processed without the support of their partners or families, with the aim of minimizing patient’s distress. Silent suffering reveals the importance of communication and sharing to mitigate distress levels. Strategy: Offer training to caregivers on the disease management, fostering communication and support to reduce anxiety and distress levels. This would help to face changes in lifestyle resulting from their role as caregivers.
Pereira et al., 2020 [46]	Assess the relation between sociodemographic, clinical, and psychological variables with quality of life and the moderating role of caregivers’ age and the caregiving duration in caregivers of patients with multiple myeloma.	Portugal/Period: Non-described	Quantitative study (cross-sectional) Instrument: HADS, SSSS, CarGOQoL, CAMI, BIS, and SF-SUNS. Sample: 118 caregivers (45 men and 73 women, average age: 58.67 years)	Study shows that family caregivers are mostly women, patients’ spouses, or elder daughters. The burden in caregivers has been associated with high levels of stress and fatigue due to concern, insecurity, and social isolation. Besides, most caregivers possess no skills which guarantee their own well-being. Strategies: Develop coping strategies through psychological intervention programs. These strategies have been associated with a better attitude of the caregiver, which would help to lessen the emotional impact of caregivers and improve their quality of life.
Reblin et al., 2020 [41]	(1) Describe communication quality between patients with cancer and their spouse caregivers through observation methods, and (2) evaluate the association between communication patient-caregiver and psychological and physical health.	USA/Period: Non-described	Quantitative study (prospective observational) Instrument: HADS and PSS. Sample: 81 caregivers (23 men and 58 women, average age: 64.95 years)	Communication difficulties in partner carers of patients with cancer causes discomfort in the latter, as well as emotional and physical health issues in both. Strategy: provide caregivers opportunities to express their concerns and communicate their emotional needs outside their relationships, and thus improve their well-being. Facilitate the access to support groups, reducing the possible logistic barriers that may prevent caregivers to attend.
Titler et al., 2020 [47]	Assess participants’ acceptability of the FOCUS program, a psychoeducational intervention, delivered to multiple patient–caregiver dyads in a small-group format.	USA/Period: Non-described	Mixed study Instrument: FOCUS Satisfaction Instrument. Sample: 36 caregivers (16 men and 20 women, average age: 55.9 years)	The lack of communication between patient and caregiver generates depression, anxiety, and distress issues. Strategy: Psychoeducational conducted by nurses. Participants felt the benefits of being able to speak their mind about their concerns and listen to other people’s thoughts, as well as the openness and ease of talking to other couples in similar situations.
Abdullah et al., 2019 [48]	Examine quality of life in relation with health in caregivers of patients with gastrointestinal cancer in combination with sociodemographic factors and other related with caregiving.	Malaysia/September 2017–February 2018	Quantitative study (cross-sectional) Instrument: CSI-M, MSPSS-M, and MCQOL. Sample: 323 family caregivers (103 men and 220 women, average age: 44.50 years)	Caregiver’s sex, ethnicity, and strain, as well as duration of caregiving provision were significantly associated with their quality of life. There was an inverse relationship among caregiving strain, duration of caregiving, and caregiver’s quality of life. Strategy: Health professionals must be able to identify groups at-risk, offering them the resources needed to improve their quality of life, including psychological therapy and access to support groups.
Baudry et al., 2019 [33]	Identify the profiles of caregivers at higher risk of having at least one moderately or highly unmet supportive care need based on socio-demographic and clinical variables highlighted in the literature.	France/18 months; year not further specified.	Quantitative study (descriptive cross-sectional) Instrument: SCNS-P&C-F, and HADS. Sample: 364 caregivers (131 men and 233 women, average age: 58.05 years)	The main problem of caregivers lies in the risk of suffering anxiety and depression depending of the type of cancer of the relative they provide care to. Likewise, caregivers show difficulty in controlling their emotional state. Strategy: Routine evaluation of anxiety and depression symptoms of caregivers to prevent risk of emotional overload.
De Camargos et al., 2019 [23]	Analyze and compare what oncologic patients, non-professional caregivers, and healthy population think could make them happy.	Brazil/October 2015–October 2016	Qualitative study through interviews. Sample: 126 caregivers (28 men and 98 women, average age: non-described)	Among problems or concerns suffered by caregivers, there is risk of depression, anxiety, and distress. Strategy: Develop psychoeducational and cognitive-behavioral strategies which help to improve the caregivers’ quality of life. These strategies should be addressed to cope with emotional overload derived from caregiving, among other concerns.
Hsu et al., 2019 [49]	(1) Characterize non-professional caregivers of hospitalized older adults with cancer, (2) determine the caregiver’s quality of life, and (3) identify the factors associated with a worse quality of life of the caregiver.	USA/July 2013–January 2014	Quantitative study Instrument: CQOLC, CCI, MHI-18, MOS, MOS-SSS, OARS-IADL, MOS-physical health scale, and KPS. Sample: 100 family caregivers (28 men, and 72 women, average age: 65 years)	Most caregivers qualified their own health as good vs a lower percentage which referred a worsening of their health condition due to caregiving. Caregivers performed a median of 35 h of care per week. A lower quality of life in the caregiver was associated with worse mental health, less social support, and worse score in both Karnofsky and patient scales. Strategy: Develop strategies, including training, skills, and guidance on how to provide care to patients, to improve the quality of life of the caregiver, their physical well-being and self-efficacy.
Kehoe et al., 2019 [42]	Evaluate the relationships between aging-related domains captured by geriatric assessment for older patients with advanced cancer and caregivers’ emotional health and quality of life.	USA/October 2014–April 2017	Quantitative study (cross-sectional) Instrument: Geriatric Scale, PHQ-2, GAD-7, and SF-12 Sample: 411 family caregivers (101 men and 310 women, average age: 66.5 years)	Almost half of the caregivers described feelings of distress, anxiety, and depression. A greater decline in the patients’ geriatric scale was associated with higher levels of depression and worsening of physical health and quality of life of caregivers. Besides, when the caregiver was younger and had greater number of comorbidities, their quality of life were worse.
Kilic y Oz, 2019 [24]	Assess quality of life of family caregivers of patients with cancer in Türkiye.	Türkiye/Period: Non-described	Quantitative study (descriptive) Instrument: QoL-FV. Sample: 378 family caregivers (135 men and 243 women, average age: non-described)	81% of the sample reported that the disease process affected their lives negatively, particularly the social and work spheres. There were significant differences between the quality of life of caregivers and their sex, education level, work situation, income level, relation with patients, and if they lived with their patients or not. Strategy: Develop strategies addressed to coping, knowledge and communication, physical activities, and search of support networks. Health professionals should conduct their strategies with an approach focused on the whole family.
Kristanti et al., 2019 [25]	Explore the experiences of family caregivers of patients with cancer in the performance of their care provision in Indonesia.	Indonesia/July 2015–March 2016	Qualitative study through interviews. Sample: 24 caregivers (8 men and 16 women, average age: non-described)	Most caregivers reported a physical impact resulting from their role of caregiver, with feelings of fatigue or a tendency to ignore their condition. Due to constant concern, fear, and permanent state of alert, there was a significant impact at an emotional level. Regarding their economic status, both families with low incomes and those of higher income suffered the consequences of the need of providing the best care. At a social level, some positive changes were reported, such as the acquisition of new values and greater family cohesion.
Shin et al., 2019 [43]	Explore the association between the needs of health care and quality of life of family caregivers of people with cancer in Korea, according to time lapse after cancer diagnosis.	South Korea/June 2014–April 2017	Quantitative study (cross-sectional) Instrument: CNAT-C and EQ-5D-3L. Sample: 686 caregivers (239 men and 447 women, average age: 49 years)	Female caregivers or older caregivers had a lower quality of life and greater unmet needs (at the health/psychological problems and religious/spiritual support dimensions) in questionnaires when compared to results of male or younger caregivers.
Steel et al., 2019 [37]	Examine psychosocial and behavioral predictors of metabolic syndrome in caregivers of patients with cancer.	USA/November 2016–August 2018	Quantitative study (prospective) Instrument: CES-D, PSS, CQOLC, Cook-Medley Hostility Scale, PSQI, IPAQ, Substance use questionnaire, The Revised UCLA Loneliness scale, ISEL, DAS-7. Sample: 104 caregivers (24 men and 80 women, average age: 59.5 years)	Almost half of caregivers included in the study met the criteria for metabolic syndrome, showing with above average figures than that of the general population. The study did not associate depressive syndrome and metabolic syndrome. Strategy: Design and test adapted strategies addressed to specific health behaviors, as well as psychological factors (caregiver’s quality of life) to reduce metabolic anomalies in caregivers of patients with cancer.
Van Roij et al., 2019 [54]	Explore the social consequences of advanced cancer in patients and non-professional caregivers.	Netherlands/January–June 2017	Qualitative study through focal groups. Sample: 15 caregivers (6 men and 9 women, average age: 58 years)	Caregivers described difficulties in reconciling their role as caregiver with the rest of daily life activities. Besides, they described abandonment of social spheres due to lack of time/energy, or feelings of shame/guilt for letting patients in other people’s care. Caregivers described feeling uneasy at social events, as most interactions were focused on cancer. Strategy: Help caregivers to express their feelings with respect to social situations through both psychological support and social awareness. Additionally, raise awareness among health professionals with respect to cancer social impact dimension.
Wittenberg et al., 2019 [26]	Explore the differences in the domains of health literacy among family caregiver communication types.	USA/March 2018–June 2018	Quantitative study (descriptive) Instrument: FCCT, HLCS-C Sample: 115 caregivers (38 men and 77 women, average age: non-described)	There were significant differences in the domains of health literacy among family caregiver communication types regarding cancer discussion with the patient and general understanding of health system. A lack of communication regarding the disease on the part of the caregiver has been positively associated with depression in the caregiver. Strategy: Develop communication and cultural awareness skills in the care provider to ensure a quality communication.
Wood et al., 2019 [55]	Quantify the humanistic burden associated with caring for patients with advanced non-small cell lung cancer from the caregiver’s perspective, including quality of life, in three different European countries.	France, Germany, and Italy/May 2015–June 2016	Quantitative study (multicenter, cross-sectional) Instruments: EQ-5D-3L, Zarit Burden Interview y WPAI:GH Sample: 427 caregivers (120 men and 307 women, average age: 53.5 years)	There were significant differences in the results of EQ-5D-3L questionnaire among caregivers receiving first-line therapy and later lines of therapy. Caregivers receiving the later lines rated their own health status significantly worse than caregivers receiving first-line therapy. General work impairment was considerable among employed caregivers.
Bilgin y Gozum, 2018 [27]	Identify the needs of nursing care given at home of both patients with stomach cancer and their caregivers, as well as the effect of family nursing care in the quality of life of both patients and their families.	Türkiye/Period: Non-described	Experimental controlled trial. Instrument: QLQ-C30, CQOLC Sample: 72 caregivers (27 men and 45 women, average age: non-described)	The assessment of the quality of life showed that caregivers presented significant changes in the dimensions of psychological strain, disruption in daily life, and care responsibility. There were no significant changes in the subscale of financial concerns. Strategy: Home care performed by nurses and focused on lack of knowledge, diagnosis of despair, anxiety, isolation/social decline. Care provided improved the scores of caregivers’ quality of life when compared to baseline situation.
Cho et al., 2018 [28]	Compare depression prevalence in relatives of patients with cancer and general population.	South Korea/2007–2014	Quantitative study Instruments: Korea National Health and Nutrition Examination Survey. Sample: 1590 caregivers (811 men and 779 women, average age: non-described)	The odds of having medically diagnosed depression in caregivers of patients with cancer were significantly higher than those of the general population. Strategy: Invest more effort in the diagnosis and management of depression in families of patients with cancer in order to improve their quality of life and the patient’s well-being.
Cubukcu, 2018 [44]	Assess quality of life and factors affecting caregivers of patients with cancer who receive care at home.	Türkiye/February de 2014	Quantitative study (cross-sectional descriptive) Instrument: CQOLC, Katz index, and Lawton index. Sample: 48 caregivers (8 men and 40 women, average age: 50.75 years)	Caregivers describe a worsening of their health. More than 90% of caregivers declare having psychological issues, and 9% confirm suffering physical decline. More than half revealed not having time to fulfill their responsibilities due to caregiving process. Quality of life is lower when caregivers are family, there is a lack of social insurance, care provision exceeds the year of duration, and income is low. Strategy: Personal situations of caregivers must be considered, analyzing their needs, and offering them both material and spiritual support.
Cuthbert et al., 2018 [34]	Examine the effects of a 12-week exercise program on the quality of life, psychological outcomes, physical activity levels, and physical fitness in caregivers of patients with cancer.	Canada/May 2015–February 2016	Randomized controlled trial (case and control) Sample: 100 caregivers (39 men and 61 women, average age: 53.25 years)	Family caregivers of patients with cancer are at risk of suffering greater physical and psychological morbidity as a result of their role as caregivers. Strategy: Exercise program conducted by a certified personal trainer and a kinesiology student volunteer. The strategy was effective in improving the physical condition of caregivers.
Hyde et al., 2018 [50]	Examine psychological distress specific in the partner of the patient with prostate cancer over two years, as well predictors of change.	Australia/January 2009–November 2010	Quantitative study Instrument: HADS, IES-R, Caregiver Burden scale, partner version of the Self-Efficacy for Symptom Control Inventory, two subscales to evaluate stress, 7-item Dyadic Adjustment Scale. Sample: 427 caregivers (0 men and 427 women, average age: 62.8 years)	Female caregivers of patients with prostate cancer referred significant psychological distress with high levels of anxiety and depression. Partners with greater burden in their role as caregiver showed greater psychological tension in comparison with those having less. More self-efficacy on the part patient was associated to less psychological discomfort in the caregiver. Strategy: Increase information on the disease process and care at home, provide practical and emotional support to caregivers, offering them different resources such as social network support, and strengthen their psychological resilience.
McDonald et al., 2018 [29]	Conceptualize quality of life of caregivers from their own perspective and explore the differences in themes between those who did or did not receive an early palliative care intervention.	Canada/December 2006–February 2011	Qualitative study through semi-structured interviews Sample: 23 caregivers (7 men and 16 women, average age: non-described)	Caregivers felt insecure about how to face the end of life of relatives they provided care to, as they felt they lacked knowledge. Strategy: Support on palliative care conducted by specialized doctors and nurses. They shared an open discussion on end of life, trying to balance hope and reality, and increased confidence from a range of professional supports.
Tan et al., 2018 [51]	Explore the interrelations between care burden, emotional state, and quality of life of caregivers of patients with lung cancer and research the association between health results of both caregiver and patient.	England/Period: Non-described	Quantitative study (exploratory cross-sectional) Instrument: CBS, CQOLC, HADS and LCSS. Sample: 43 caregivers (15 men and 28 women, average age: 61.7 years)	46.5% caregivers were identified as anxious and 27.9% as depressed. Those caregivers experiencing anxiety and depression scored worse quality of life and higher burden when compared with those non-anxious and non-depressed caregivers. Depressive emotional state in patients seemed to be associated with higher emotional distress in caregivers.
Washington et al., 2018 [38]	Examine both viability and impact of problem-solving therapy for caregivers of patients with cancer receiving outpatient palliative care.	USA/October 2015–February 2017	Randomized controlled trial Instrument: PST, GAD-7, PHQ-9, CQLI-R Sample: 83 caregivers (26 men and 57 women, average age: 51.5 years)	Caregivers described high psychological strain tension and high levels of anxiety and depression which directly affected their quality of life. Strategy: Problem-solving therapy conducted by nurses. Participants informed of lower anxiety levels. However, no significant differences were observed regarding caregivers’ depression or quality of life.
Yu et al., 2018 [39]	Evaluate quality of life related to the health of family caregivers of patients with leukemia using the health-related utility scores derived from questionnaire EQ-5D.	China/July 2015–February 2016	Quantitative study (cross-sectional) Instrument: EQ-5D-3L, HADS, SSRS, family APGAR Sample: 306 caregivers (139 men and 167 women, average age: 41.20 years)	Caregivers showed lower scores in EQ-5D-3L questionnaire than general population. Participants of a lower socioeconomic status had lower scores and reported more problems that those with a higher socioeconomic status. Strategy: Offering and promoting both financial and social support may be key to improve the quality of life of family caregivers.
Lee et al., 2017 [52]	Explore the mood changes and quality of life in caregivers of patients with head and neck cancer and examine the prevalence and risk factors of depressive disorders among caregivers of patients suffering this type of cancer.	Taiwan/February 2012–January 2013	Quantitative study (prospective) Instrument: SCID-CV, HADS, SF-36, and Family APGAR Index. Sample: 132 caregivers (30 men and 102 women, average age: 47.2 years)	During the 6-month follow-up, depression and anxiety severity in caregivers decreased, which significantly improved their quality of life. After six months, most prevalent psychiatric disorders were depression-related, followed by alcohol abuse and primary insomnia. Study revealed that older ages, the use of hypnotic drugs, pre-existing depression, and lower mental component of SF-36 score at baseline were found to significantly predict depressive disorders in caregivers of patients with these types of cancer.
Mollica et al., 2017 [30]	Examine association between the receipt of medical/nursing skills training and the caregiver burden as well as the mediation of caregiving confidence on this relationship.	USA/Period: non-described	Quantitative study (cross-sectional) Instrument: ZBI (short version), 2 questions to measure receipt of medical/nursing skills and 1 question to measure the caregiver’s training. Sample: 641 caregivers (125 men and 516 women, average age: non-described)	Caregivers reported moderate levels of burden. Lack of receipt of training was associated with greater levels of burden in their role as caregivers. Confidence partially mediated the relation between training and burden. Strategy: Authors highlighted the need of empirical studies which evaluate if training in medical/nursing skills for caregivers of patients with cancer may have an impact in the outcomes of caregivers over time.
Senneseth et al., 2017 [31]	(1) Measure the short-term effects of the Cancer-PEPSONE received social support, psychological distress, and quality of life of partners, and (2) explore social support received as a mediator of the intervention effects.	Norway/ December 2013–June 2015	Open single-center randomized controlled trial. Instrument: CSS, MSPSS, GHQ-12, QOLS-N Sample: 35 caregivers (21 men and 14 women, average age: non-described)	The control group referred a significant decrease in perceived social support. According to this study analysis, CPP may have indirect effects in the short term on the caregivers’ quality of life through social support received. Strategy: The intervention group received a 3-h session. Evaluation: Firstly, psychoeducative aspects analyzing challenges associated to the disease and social support. Then, individual needs of each family were analyzed, exploring the resources required for each case.

BIS: Burden Interview Scale; Brief-POMS: Brief-Profile of Mood States; CAMI: Community Attitudes to Mental Illness; CarCOQol: Caregiver Oncology Quality of Life questionnaire; CASE-T: Cancer Self-Efficacy Scale-Transplant; CBS: Caregiver Burden Scale; CCI: Charlson Comorbidity Index; CES-D: Center of Epidemiological Studies of Depression Scale; CNAT-C: Comprehensive Needs Assessment Tool for Cancer Caregivers; CQLI-R: Caregiver Quality of Life Index-Revised; CQOLC: Caregiver Quality of Life Index-Cancer; CRA: Caregiver Reaction Assessment; CSI-M: Caregiver Strain Index-Malay; CSS: Crisis Support Scale; DAS-7: 7-Item Dyadic Adjustment Scale; EQ-5D-3L: EuroQol five-dimension three-level; FACT-G: Functional Assessment of Cancer Therapy—General; FCCT: Family Caregiver Communication Tool; GAD-7: Generalized Anxiety Disorder-7; GHQ-12: The 12-Item General Health Questionnaire; HADS: The Hospital Anxiety and Depression Scale; HLCS-C: Health Literacy of Caregivers Scale—Cancer; IES-R: Impact of Event Scale—Revised; IPAQ: International-Physical-Activity-Questionnaire; ISEL: Interpersonal Support Evaluation List; KPS: Karnofsky Performance Scale; LCSS: Lung Cancer Symptom Scale; MCQOL: Malay Caregiver Quality of Life scale; MCSDS: Marlowe-Crowne Social Desirability; MHI-18: Mental Health Inventory-18; MOCS: Measure of Current Status; MOS: Mean Opinon Scale; MOS-SSS: Medical Outcomes Study Sleep Scale; MSPSS: Multidimensional Scale of Perceived Social Support; MSPSS-M: Malay Multidimensional Scale of Perceived Social Support; OARS-IADL: Older Americans Resources and Services Scale—Instrumental Activities of Daily Living; PHQ: Patient Health Questionnaire; PSQI: Pittsburgh Sleep Quality Index; PSS: Perceived Stress Scale; PST: Personnel Selection Test; QLQ-C30: Quality of Life Questionnaire-Cancer 30; QoL-FV: Quality of Life-Family Version; QOLLTI-F: Quality of Life in Life Threatening Illness-Family Carer version; QOLS-N: Norwegian version of the Quality of Life Scale; SCID-CV: Structured Clinical Interview for Disorders—Clinical Version; SCNS-P&C-F: Psychometric validation of the French version of the Supportive Care Needs Survey for Partners and Caregivers of cancer patients; SF-SUN: Short Form- Survivor Unmet Needs Survey; SF: Short Form; SSRS: The Columbia-Suicide Severity Rating Scale; SSSS: Social Support Satisfaction Scale; UWQOL: University of Washington Quality of Life questionnaire; WPAI:GH: Work Productivity and Activity Impairment Questionnaire: General Health; ZBI: Zarit Burden Interview.

## 4. Discussion

### 4.1. Characteristics of the Sample of Caregivers of Adults with Cancer

The total sample was made up of 7663 caregivers, 66.72% were women. In this sense, most studies coincide in stating that there are more women caregivers of patients with cancer than men [22,23,24,25,26,27,29,30,32,33,34,35,36,37,38,39,40,41,42,43,44,45,46,47,48,49,50,51,52,53,54,55,56,57]. This data met the results obtained by other authors outside this review, such as Martínez et al. [58] who conducted research to define the burden of caregivers of patients with head and neck cancer, concluded that the profile consisted of 81% women. On the other hand, the study accomplished by Guijarro-Requena et al. [13], with the aim of improving the quality of life of caregivers through training initiatives, indicated as well a larger representation of women, reaching 91.9% of the sample. However, there were two studies that described a larger number of male caregivers with respect to women [28,31].

Regarding average age of caregivers of patients with cancer, most of the studies set the interval between ages 45–65 [32,33,34,35,36,37,38,40,41,43,44,45,46,47,48,49,50,51,52,54,55,56]. These results coincide with those described by other authors [59,60]. Notwithstanding, in the study by Peredo et al. [61] analyzing burnout syndrome, anxiety, and depression in caregivers of patients with cancer, 48.7% were among the age interval 20–40 years. In the study by Amador et al. [62] analyzing psycho-affective features and overburden levels in caregivers of oncologic patients, 48% were women aged 24–29 years.

With respect to the familiar bond, the role of caregiver is undertaken by spouses or daughters/sons of people with cancer and usually share the same home [22,23,25,26,27,28,30,32,33,34,35,36,37,38,39]. These results are aligned with the conclusions described by other authors [5,8,58,63].

### 4.2. Physical, Emotional, Social, and Financial Strain Suffered by Caregivers of Adults with Cancer

Different review studies have revealed that care provision on the part of caregivers causes physical problems, increasing the risk of suffering physical morbidity the longer the care activity continues [25,34,37,40,41,42,43,44]. Authors outside this review described how the stressful factors related to care provision considerably affect the physical condition of caregivers, with this impact higher on male caregivers who are older and bearing a high demand of care [64]. Barrón and Alvarado [65] indicated that care provision, particularly if prolonged over time, may result in various physical conditions such as: fatigue, headaches, joint pain, dizziness, and worsening of sleep quality to trouble sleeping.

Most of the studies [22,23,25,26,27,28,29,30,33,34,35,36,38,40,41,42,43,44,45,46,47,48,49,50,51,52] have referenced the emotional and psychological problems suffered by caregivers, describing feelings of distress, stress, high levels of anxiety, and even depression. Emotional strain was significantly higher when care provision extended over time. Results obtained are in line with those mentioned by other authors [64,66]. Besides, the study conducted by Rosado et al. [67], aimed to evaluate the support needs and quality of life of caregivers of pediatric patients with cancer, described that emotional performance was the most damaged in the total score of quality of life index. 

Regarding social problems, review studies [24,25,27,31,32,41,49,53,54,55] described how social interactions of caregivers were affected to the point of finding it very difficult to reconcile their role of caregivers with the rest of activities, where deterioration of working conditions and lesser social event engagement were highlighted. Caregivers who declared having worse social support showed poorer quality of life indicators. These findings are aligned with other authors’ results, which also indicated deterioration of working conditions, and a decrease of free time to develop daily activities or taking time for themselves. Moreover, the lesser the social support is perceived on the part of the caregiver, the worse the consequences on her/his health, both physical and psychological [66,68].

Several review studies [25,27,39,44,53,56,57] mentioned the financial problems of caregivers, highlighting among the possible causes, the lack of financial support, the costs derived from the disease process itself, or the difficulty of developing their work activity. Caregivers who reported lower incomes showed worse quality of life indicators. Several authors outside the review reported that difficulties to reconcile the role of caregiver with work activities add multiple financial burdens to caregivers [66]. The study conducted by Cortijo-Palacios et al. [69] carried out the Zarit Burden Interview in assessing caregiving burden, revealing high scores in low spending capacity and feelings of financial distress. 

### 4.3. Strategies to Improve the Quality of Life of Caregivers of Adults with Cancer

With respect to the strategies to improve the quality of life of caregivers, different studies have pointed out the need of promoting social support, facilitating the access to support networks [28,29,31,32,36,39,41,50,54,57]. Authors such as Martínez, Lorenzo, and Llantá [58] have indicated that in order to improve the quality of life of caregivers, both social and family support are key, as they not only allow to hand over some tasks and responsibilities, but they also provide emotional, spiritual, and material support. 

Review studies have described the need of implementing coping strategies to allow patients to improve the management of their own disease, while developing skills to reduce the emotional and physical impact [23,24,27,30,32,34,35,37,38,45,46,47,49,53,54]. These findings coincide with those published by other authors such as Pino et al. [70] who described the need of implementing coping strategies to protect the quality of life of caregivers and thus overcome cognitive, emotional, and behavioral demands faced by both caregiver and family environment. Besides, Pérez et al. [71] indicated that caregivers of patients with cancer who were able to develop coping strategies, managed to decrease the emotional impact and fatigue feeling. 

Review studies revealed the need of appraising the economic situation of caregivers [25,27,39,44,53,56,57]. Authors such as Carreño et al. [72] have also pointed out this need when facing the high economic burden imposed on caregivers, and that this is not only associated to medical services, but to the lack of labor productivity or even job loss. This financial burden is connected to higher levels of anxiety and distress suffered by caregivers.

Authors of the review pointed out that in order to develop the strategies to aid caregivers, it is necessary to properly train health professionals, both in the management of emotional and physical strain, as in covering the spiritual needs and facilitate access to support networks. This requires health professionals to able to identify the most vulnerable groups while considering providing specific health care to all the family [22,24,44,48]. Moreover, in order to comply with all these needs, health professionals require cultural awareness to understand better the complex requirements of caregivers [26]. These results are aligned with the findings described by other authors [73].

### 4.4. Limitations

This review shows some limitations. Shortening the search strategy to the last five years made it impossible to recover all the information available on this subject, though a rigorous process was followed in order to obtain the latest scientific findings. Furthermore, the number of subjects in the sample vary from one study to another. On the other hand, the diversity of the research designs that were employed also limits interpretations. Another limitation is that no tool was used for study quality assessment.

## 5. Conclusions

The vast majority of caregivers of adults with cancer are women around 50 years old, being most commonly spouses or daughters of the patient with whom they usually live. Caregiving generates physical, emotional, social, and financial problems, which cause a burden in caregivers that results in a decrease of their quality of life. To improve their quality of life, different strategies can be implemented: In relation to physical problems, it is recommended exercising practice to improve the perceived health levels on the part of caregivers. At the emotional level, it is recommended, on one hand, enhancing social support by means of reinforcing the support networks and facilitating access to support groups and associations; and, on the other hand, developing coping strategies which improve the management and control of both the situation and the emotional strain. Breathing exercises and counseling sessions to reduce emotional strain have also been described is this regard. In the economic sphere, it stands out the need to provide financial support to caregivers in order to improve their well-being. In order to implement these strategies, it is necessary to train health professionals.

## Data Availability

Not applicable.
